# Pyogenic Liver Abscess and the Important Role of Point-of-Care Ultrasound (POCUS) in Daily Practice: A Report of Two Cases

**DOI:** 10.7759/cureus.72444

**Published:** 2024-10-26

**Authors:** Abdur Rahman Rubel, Babu Ivan Mani, Vui H Chong

**Affiliations:** 1 Department of Medicine, Pengiran Muda Mahkota Pengiran Muda Haji Al-Muhtadee Billah (PMMPMHAMB) Hospital, Tutong, BRN; 2 Department of Medicine, Raja Isteri Pengiran Anak Saleha (RIPAS) Hospital, Bandar Seri Begawan, BRN

**Keywords:** diagnosis, liver abscess, pocus, transabdominal ultrasound, ultrasound

## Abstract

A pyogenic liver abscess (PLA) is uncommon and a potentially life-threatening condition. Clinical manifestations and laboratory investigations can be non-specific and the detection of PLAs requires imaging, which can often be delayed. Point-of-care ultrasound (POCUS) is now becoming more widely adopted and plays an important role in clinical practice. We report two cases of PLA, one of which was a gas-forming PLA that we encountered in a district general hospital where POCUS played an important role in the diagnosis and management. The diagnoses of PLAs were initially unsuspected due to a combination of non-specific symptom manifestations, initial negative imaging, and a subtle radiological clue that was missed due to a lack of awareness. Bedside POCUS examinations were done due to clinical deterioration in one patient and lack of inflammatory marker improvement in both patients. These cases highlight the important role of POCUS in the management of patients with liver abscesses.

## Introduction

Infection remains an important cause of hospitalizations and mortality globally. A pyogenic liver abscess (PLA) can be considered relatively uncommon but is increasing, and though the rates have gone down, it is still associated with a mortality rate of between 2% and 12% [1]. Gas-forming PLA (GFPLA) is an uncommon manifestation of PLA and is associated with a higher mortality rate [2]. Commonly implicated bacteria include *Klebsiella pneumoniae* and *Escherichia coli*. Management requires early detection and appropriate treatment with antibiotics and in some instances drainage, either percutaneous or surgical [1]. Early diagnosis is therefore very important. However, clinical and laboratory investigations can be non-specific and result in a delayed diagnosis. A definitive diagnosis always requires imaging. A point-of-care ultrasound (POCUS), which is portable and can be done at the bedside providing instant information, is now increasingly being adopted in clinical practice. We report two cases of PLA, one of which was a GFPLA, where POCUS played a central, important role in the detection of PLA.

## Case presentation

Case 1

A 62-year-old female presented to the Emergency Department with a two-day history of productive cough with whitish sputum, mild dyspnea, reduced appetite, and non-vertiginous dizziness. She also had transient, mild, non-specific right upper abdominal discomfort that had settled by the time of admission to the ward, after being given analgesia (50 mg intramuscular tramadol) and intravenous omeprazole (40 mg bolus) in the emergency department. She denied any chest pain or urinary or other gastrointestinal symptoms. Her past medical history was relevant for poorly controlled diabetes mellitus (latest glycated hemoglobin (HbA1c) 15.3%) and dyslipidemia. Physical examination revealed a temperature of 37.7 degrees Celsius and otherwise, not in distress. Respiratory examination only revealed bilateral crepitations at the lung bases. There was no abdominal guarding but the abdomen was mildly tender on deep palpation of the upper abdomen. The rest of the examinations (neurological and musculoskeletal) were normal. Blood investigations showed elevated glucose of 405 mg/dL or 22.5 mmol/L but were negative for serum ketones, elevated C-reactive protein (CRP), lactate (3.4 mmol/L, normal range 0.5-2.2), and neutrophil-predominant leukocytosis (Table 1). Liver and renal profiles were normal. Urinalysis was negative for nitrite but demonstrated significant bacteria. Chest radiography (CXR) was reported as normal. Table 1 shows the laboratory investigation changes over the course of hospitalization.

**Table 1:** Inflammatory and liver profiles for Case 1 at admission, diagnosis (*) of GFPLA, and discharge GFPLA: gas-forming pyogenic liver abscess

	Patient results						
Laboratory investigations	Day 1	Day 2	Day 3	Day 4 (*)	Day 5	Day 42	Reference range
Inflammatory profiles							
White cell count	17.6	17.8	-	17.6	16.1	11.4	4.2-12.6x10^3^/uL
C-reactive protein	12.8	27.3	-	24.0	18.7	0.7	0.0-0.5 mg/dL
Procalcitonin	-	2.72	35.2	25.2	-	-	<0.5 ng/ml low risk for severe/ or septic shock: >2.0 ng/mL high risk and/or septic shock
Liver profiles							
Bilirubin	11.9	11.2	-	7.1	7.9	10.3	0-21 umol/L
Alanine aminotransferase	20	39	-	63	50	14	10-35 U/L
Alkaline phosphatase	84	90	-	105	99	139	35-104 U/L
Gamma-glutamyl transferase	69	82	-	110	117	214	5-36 U/L
Albumin	38	38	-	31	26	36	35-52 g/L
Total protein	73	73	-	65	57	70	66-68 g/L

**Figure 1:**
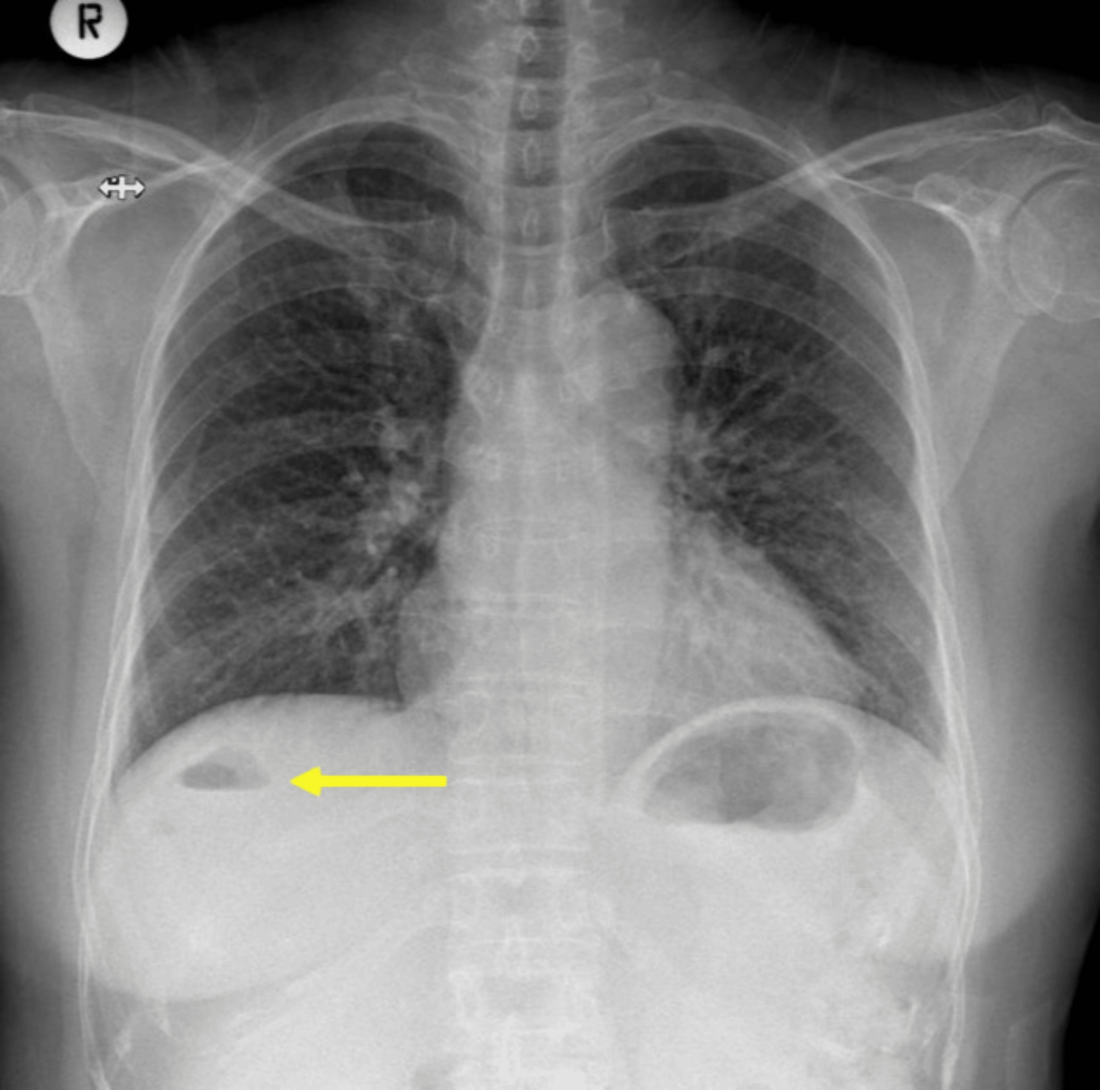
Admission chest radiograph showing normal lung fields but also air pockets (arrow) in the right upper abdomen in the position of the liver that was missed

Though the CXR was normal, the patient was treated for a probable lower respiratory tract infection based on the history of cough and dyspnea and chest examination findings. Intravenous amoxicillin-clavulanic acid (1.2 g three times a day) and subcutaneous insulin therapy for hyperglycemia were initiated. The patient's condition improved but she continued to have temperature spikes. The patient also developed diarrhea after starting antibiotic therapy but *Clostridium difficile* testing was negative. On the evening of the third day of admission, the patient had a higher fever spike with worsening of procalcitonin (Table 1). A bedside POCUS the following day using a repurposed echo machine showed a large, poorly demarcated, predominantly hypoechoic cavity located near the dome of the liver with hyperechogenic foci with mild posterior reverberation artifacts, consistent with a GFPLA. Interestingly, a review of the admission chest radiograph showed abnormal gas pockets in the liver (Figure 1).

**Figure 2:**
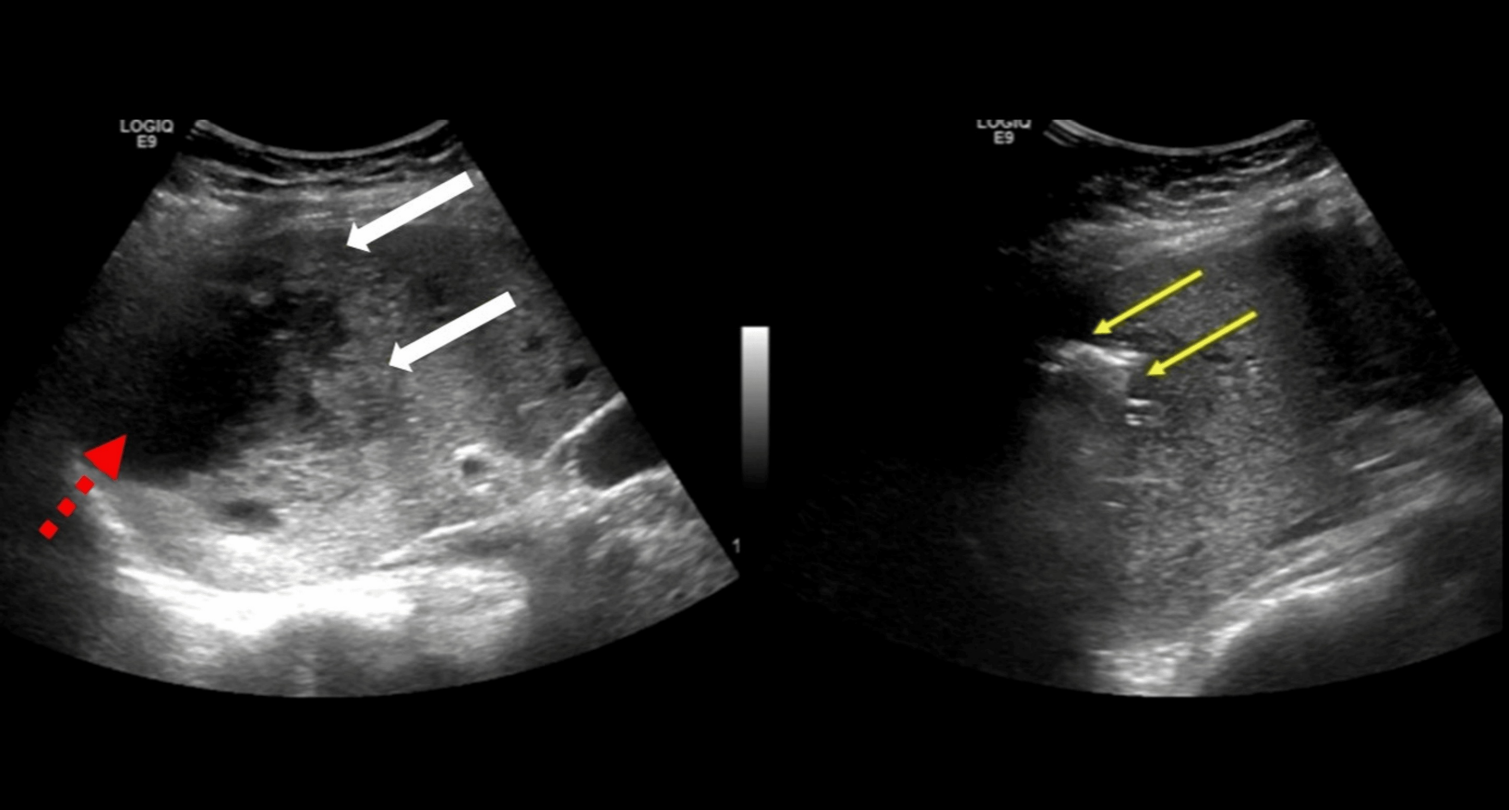
Ultrasound scan of the abdomen showing a large irregular hypoechogenic cavity (red broken arrow) with a solid component at the boundary (white arrows) located in the dome of the diaphragm. A different section of the lesion showed irregular echogenic shadows with mild posterior reverberation artifacts (yellow arrows) of air collections consistent with a GFPLA. GFPLA: gas-forming pyogenic liver abscess

The antibiotic was changed to meropenem. The patient proceeded to a formal departmental ultrasound scan, which confirmed the POCUS findings (Figure 2), and a diagnosis of GFPLA affecting liver segments 7 and 8 was made.

**Figure 3:**
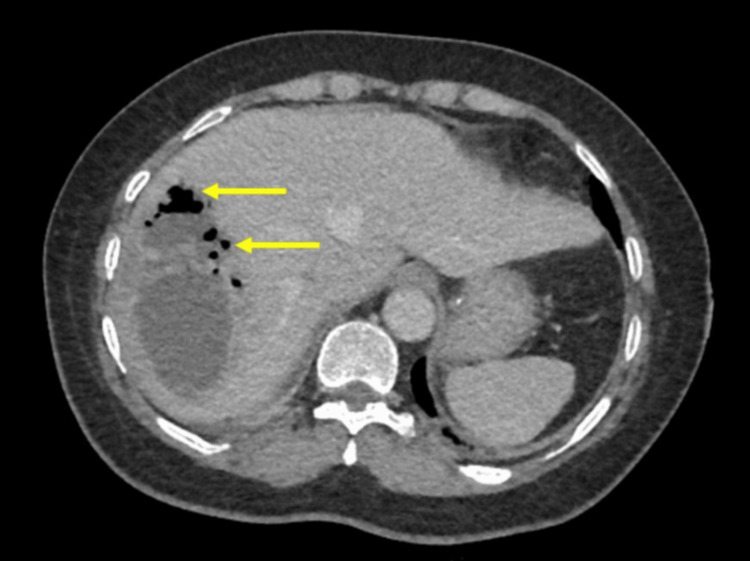
An axial contrast computed tomography image showing a large liver abscess with pockets of gas (arrows) consistent with a GFPLA GFPLA: gas-forming pyogenic liver abscess

Sputum and blood cultures revealed *Klebsiella pneumoniae*, sensitive to meropenem. The inflammatory markers improved slowly but the liver profiles started to become more abnormal (Table 1). A computed tomography (CT) scan with contrast was done when the radiologist was on-site and this showed a large (12 x 7 cm) GFPLA (Figure 3). In addition, there were gallstones and common bile duct dilatation with a stone at the distal end. 

In view of these findings, the patient was transferred to a tertiary care center for endoscopic retrograde cholangiography pancreatography (ERCP) and abscess drainage. Following the transfer, she proceeded with an ERCP stone clearance followed by the placement of a biliary stent. After ERCP and 9 days of meropenem, with the patient improving, the antibiotic was de-escalated to intravenous ciprofloxacin (400 mg twice daily) as advised by the infectious disease team. The infectious disease team also advised to refer the patient to the ophthalmology department to assess for endophthalmitis, and this was normal.

The patient completed a total of six weeks of intravenous antibiotics and was continued with oral ciprofloxacin (500 mg twice daily) for another two weeks. She was discharged and was followed up with laboratory and clinic monitoring with POCUS which showed resolution of the GFPLA.

Case 2

A 43-year-old male with a past medical history of chronic hepatitis B and dyslipidemia was admitted with a five-day history of intermittent fever. This became persistent and was associated with chills and rigors on the day of admission. The patient also complained of mild intermittent epigastric discomfort and reduced appetite. Six days prior, the patient had presented to the general practitioner and was treated with oral amoxicillin for acute tonsillitis. On examination, there was a fever of 39 degrees Celsius, mild tachycardia (heart rate 110/minute), and mild epigastric tenderness without guarding. The remainder of the physical examination revealed no obvious abnormalities. Investigations revealed elevated inflammatory markers (CRP, leukocytosis, and procalcitonin) and slightly abnormal liver profiles. Table 2 shows the laboratory investigations over the course of hospitalization.

**Table 2:** Inflammatory and liver profiles for Case 2 at admission, diagnosis (*) of PLA, and discharge PLA: pyogenic liver abscess

	Patient results					
Laboratory investigations	Day 1	Day 2	Day 3	Day 4 (*)	Day 19	Reference range
Inflammatory profiles						
White Cell Count	17.2	-	16.3	16.3	4.3	4.2-12.6x10^3^/uL
C-reactive protein	23.0	-	19.5	17.9	0.5	0.0-0.5 mg/dL
Procalcitonin	7.24	-	-	-	-	<0.5 ng/ml low risk for severe/or septic shock: >2.0 ng/mL high risk and/or septic shock
Liver profiles						
Bilirubin	22.0	-	14.3	13.2	5.6	0.0-21.0 umol/L
Alanine aminotransferase	75	-	66	65	21	10-50 U/L
Alkaline phosphatase	227	-	202	241	180	40-129 U/L
Gamma-glutamyl transferase	163	-	135	171	202	10-71 U/L
Albumin	33	-	28	28	35	35-52 g/L
Total protein	71	-	61	66	82	66-87 g/L

**Figure 4:**
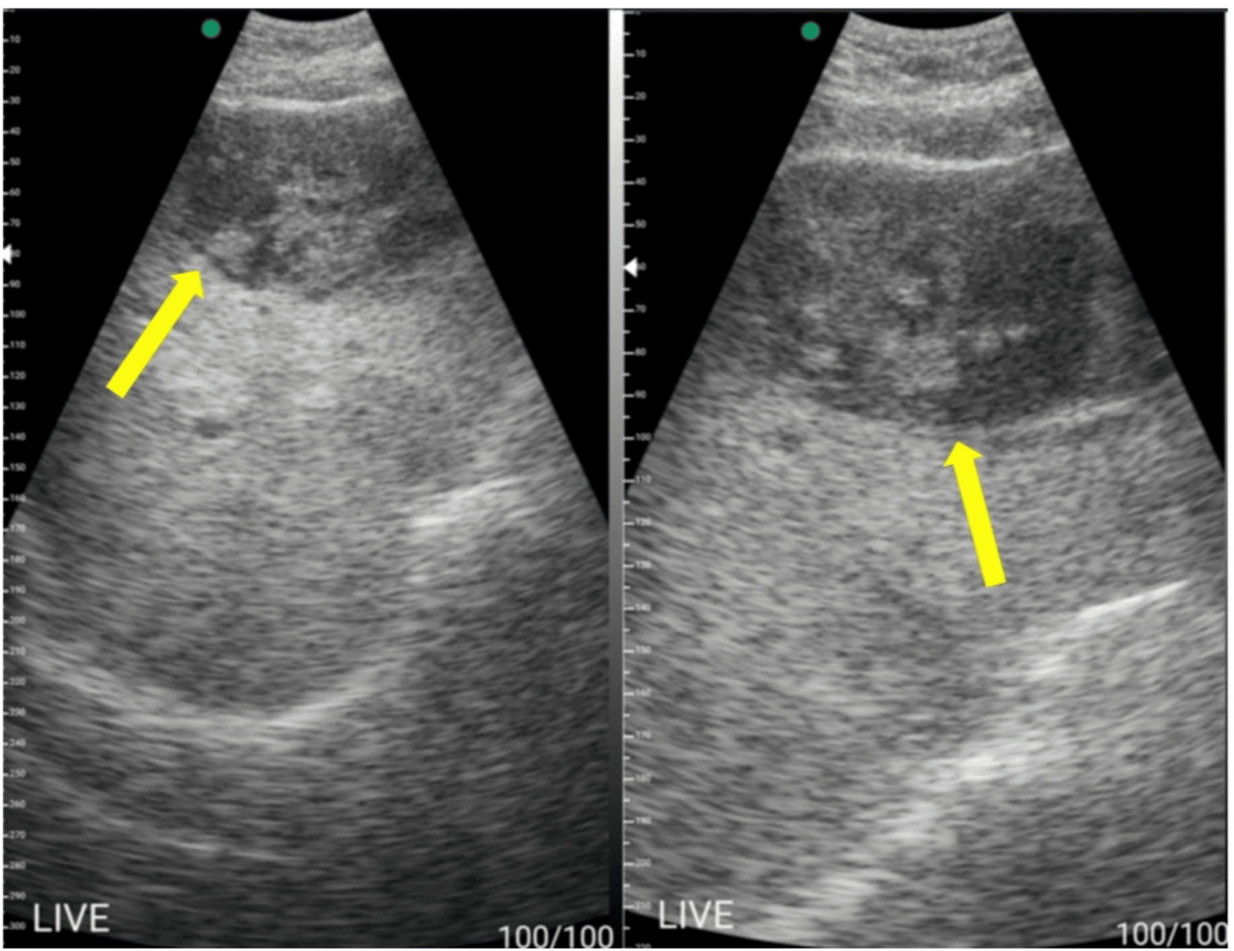
POCUS images using a portable handheld ultrasound machine showing a large mixed hypoechoic cavity (yellow arrows) with internal debris affecting the left lobe of the liver, segments 2 and 3 POCUS: point-of-care ultrasound

CXR and abdominal radiographs were normal. A departmental ultrasound scan of the abdomen was also done on the day of admission, and this only showed mild fatty liver and a slightly edematous gallbladder wall without any gallstones. He was treated for acalculous chronic cholecystitis and was started on intravenous ceftriaxone (1 g twice daily). Despite the resolution of symptoms, there was only a slight improvement in the CRP (17.9 mg/dL) and his liver profile remained abnormal (Table 2). A bedside POCUS using a portable handheld machine was done and this detected a large mixed echogenic cavity with internal debris occupying the left lobe of the liver, segments 2 and 3, consistent with a PLA (Figure 4). The hypoechogenic areas were compressible. 

**Figure 5:**
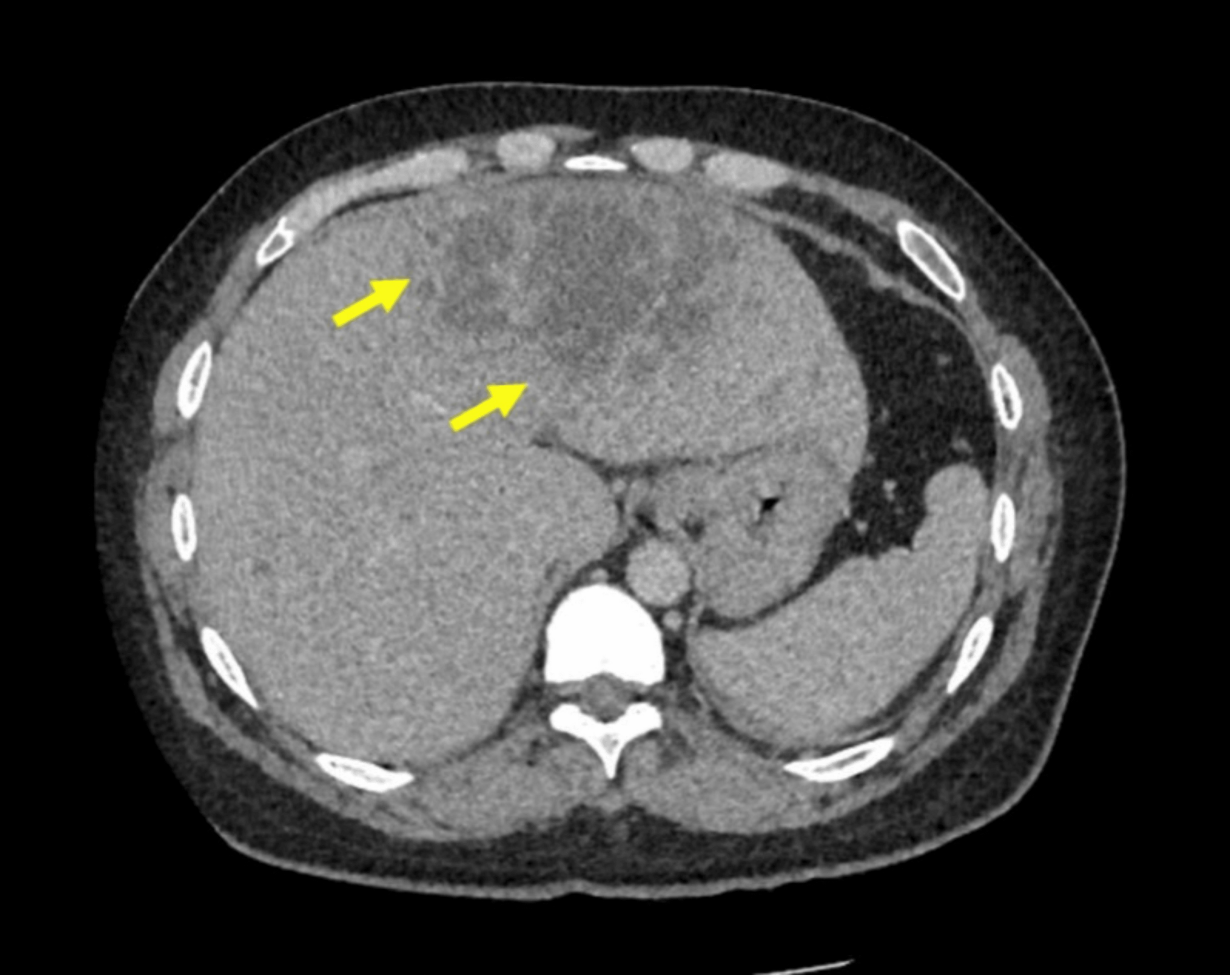
Axial CT image showing the large abscess (12 x 7 cm) indicated by the arrows corresponding to POCUS findings. The hypoechogenic areas represent liquefaction of the liver abscess. POCUS: point-of-care ultrasound

A contrast CT scan was done the following day and confirmed the findings of the POCUS (Figure 5).

In view of the size of the PLA, the patient was transferred to a tertiary care center for drainage. A drainage catheter was placed under ultrasound guidance. Culture of the PLA aspirate isolated *Klebsiella pneumoniae* that was sensitive to ceftriaxone. As per the recommendation of the infectious disease team, a referral to the ophthalmology department was made, and this ruled out the presence of endophthalmitis.

The patient was treated with four weeks of intravenous ceftriaxone, including nine days of outpatient antibiotic therapy, followed by two weeks of oral ciprofloxacin (500 mg twice daily). The patient remained well and clinic monitoring with the laboratory and POCUS at 10 weeks showed resolution of the liver abscess.

## Discussion

We report two interesting cases of PLAs where POCUS played a central role in diagnosis and altered subsequent management. Without POCUS, the diagnoses would have been missed or delayed, which could have affected overall outcomes. In our cases, diagnoses of PLAs were made several days after admission due to initial misdiagnoses, and several factors contributed to this delay. First, the presenting symptoms, aside from abdominal discomfort, are non-specific and common to various infections. Second, although laboratory investigations, particularly inflammatory markers, indicated underlying sepsis, they are not specific to PLAs. Third, imaging abnormalities were missed, which impacted clinical decisions. The air pockets seen in the CXR in Case 1, an important clue for GFPLA, were overlooked by both the admitting clinician and the radiologist, suggesting that awareness is crucial. Interestingly, in Case 2, despite the size of the PLA, the initial ultrasound completely missed the lesion. The reason for this is not known, but it is likely that the left lobe of the liver was only briefly or not assessed during the initial ultrasound scan. POCUS examinations were performed in both cases due to the failure of inflammatory markers to improve, worsening liver function tests, or clinical deterioration. The worsening of liver function tests - mild in Case 1 and more pronounced in Case 2 - raised suspicion for an underlying liver abscess, especially when the blood culture returned positive for *Klebsiella pneumoniae* bacteremia. However, one must be aware that liver dysfunction can also occur in sepsis without PLAs, as the liver plays an important role in the host immune response to sepsis [3]. Additionally, procalcitonin levels were elevated in both patients, with a significant increase observed on the third day for Case 1, strongly indicating a bacterial infection. Consequently, a thorough search for underlying infections was essential. POCUS identification of PLAs significantly altered the course of management.

For the detection of a PLA, imaging is required, and the most commonly used modalities are ultrasound and CT scans, both typically performed in the radiology department of most healthcare institutions. With an increasing patient load, it is not unexpected to encounter delays in obtaining imaging investigations, and this applies to all settings. In low-resource environments, these imaging modalities may not be available [4]. Alternatively, even when one or both imaging modalities are accessible, these services may not be available every day. In our district hospital, which operates in a moderate-resource setting, a radiologist is on-site only three days a week, contributing to delays in obtaining formal imaging. Therefore, the availability of POCUS is crucial for mitigating diagnostic delays. POCUS was introduced into our clinical practice just prior to the first case, and its initial use was sporadic. With experience, it is now utilized daily in both inpatient and outpatient settings, supplementing the services provided by the radiology department. However, the number of clinicians experienced in POCUS remains limited at this time.

To date, there have been only a few publications highlighting the use of POCUS in PLAs [5-9]. In most of these cases, diagnoses of PLAs were made in the emergency department or soon after admission due to the immediate availability of POCUS. This led to early diagnosis and the implementation of appropriate treatment at the initial point of contact [6-9]. However, in our cases, POCUS examinations were conducted several days after admission, resulting in a delay of several days to diagnosis, demonstrating the importance of early POCUS to avoid delays in diagnosis and management. On POCUS examination, PLAs are typically poorly demarcated and exhibit a variable appearance that ranges from mostly hypoechoic (liquefaction), with or without internal echoes (debris), to hyperechoic, depending on the stage of abscess development [6]. For GFPLA, there are also ill-defined hyperechoic internal echoes with posterior reverberation artifacts due to gas pockets [5]. Interestingly, POCUS-guided interventions, such as aspiration, have also been reported, eliminating the need to arrange for formal interventions that may cause delays [5].

The use of POCUS in clinical practice is gaining acceptance and has already been incorporated into many specialties [10]. Its usefulness is evident in areas such as emergency and trauma departments for rapid assessments of intra-abdominal trauma (the FAST protocol: Focused Assessment with Sonography for Trauma), as well as in critical care and anesthesia settings for assessing respiratory conditions (the BLUE protocol: Bedside Lung Ultrasound in Emergency) and assessment of intubation [11-13]. In emergency medicine and cardiology, bedside echocardiography is well-established for the bedside assessment of cardiac function, fluid status, and pulmonary edema [14]. The value of POCUS in clinical practice was greatly highlighted during the COVID-19 pandemic, where it was used for the rapid assessment of pulmonary disease progression (e.g., lung field involvement, effusions, pneumothorax), as well as for evaluating cardiac conditions (e.g., cardiac function, pericardial effusion, or pulmonary embolism) and fluid status (e.g., assessment of inferior vena cava collapsibility) [10,15]. The use of POCUS is now gaining ground in other specialties, including pediatric surgery, gastroenterology, hepatology, nephrology, and community care [16-20].

In our setting, POCUS has changed the way we practice, as it provides immediate information that influences clinical decisions. POCUS is now used on a daily basis, supplementing laboratory investigations and complementing formal radiological assessments. Targeted POCUS scanning or systematic evaluations for possible sites of infection can typically be completed in just a few minutes. This can be done at the time of admission or during or after daily ward rounds. In cases where abnormalities are detected, formal imaging is requested to better define these abnormalities. For cases where POCUS findings are negative or uncertain, formal imaging can still be performed to avoid missing small foci of infection.

## Conclusions

Our cases highlight the importance of POCUS in the management of patients admitted with intra-abdominal sepsis, specifically PLAs. If POCUS examinations had not been conducted, the diagnoses of PLAs would have been delayed or missed, resulting in delays in appropriate treatment. This is especially true in low-resource settings where radiological imaging may not be available or may only be accessible a few days a week. The role and importance of POCUS in clinical practice are gaining acceptance, as evidenced by the increasing number of publications across various specialties.
